# Mediating effects of emotional intelligence on the relationship between empathy and humanistic care ability in nursing students: A cross-sectional descriptive study

**DOI:** 10.1097/MD.0000000000031673

**Published:** 2022-11-18

**Authors:** Meng Lina, Guan Qin, Li Yang

**Affiliations:** a Department of Nursing, Harbin Medical University, Heilongjiang, China; b Department of Education, Harbin Medical University, Heilongjiang, China.

**Keywords:** emotional intelligence, empathy, humanistic care ability, nursing students

## Abstract

Patient-centered nursing holistic care is of utmost important to the nursing profession, and humanistic care cultivation has become a global nursing education concern. This study aimed to examine the relationship among emotional intelligence, empathy and humanistic care ability in nursing students, and to determine whether positive emotional intelligence could mediate the relationship between empathy and humanistic care ability. This study used a cross-sectional, descriptive design. A total of 323 nursing students was enrolled from one medical university in Heilongjiang Province, China. The emotional intelligence questionnaires, empathy scale and humanistic care ability scale were used to quantify participants’ responses. There was no significant difference in gender, residence, single-child family and leader experience of nursing students’ humanistic care ability. However, the significant differences were found in grade (*t* = 4.55, *P* < .01) and major interests (*t* = 7.06, *P* < .01). Obviously, there was positive correlation between positive emotional intelligence and empathy (*R* = 0.37, *P* < .01), and positive correlation between humanistic care ability and emotional intelligence (*R* = 0.62, *P* < .01), and empathy (*R* = 0.57, *P* < .01). Furthermore, emotional intelligence (β = 0.21, *P* < .01) had a significant mediating effect on the relationship between humanistic care ability and empathy. Nurse educators should improve the empathy of nursing students by developing and implementing emotional intelligence programs, in order to improve their humanistic care ability.

## 1. Introduction

“Sometimes to heal, always to help, always to care” is Trudeau’s dictum that captures the main work of nursing. Humanistic care is not only the core of nursing work, but also the transmission of human nature and the embodiment of humanistic spirit.^[1]^ Humanistic care ability, as one of the core competencies necessary for qualified nurses, has become the focus of nursing education. It refers to understanding the cultural background of patients, respecting the values of patients, expressing the caring feelings of nursing staff, coordinating the interpersonal relationship of patients, and implementing personalized care according to the needs of patients.^[2]^

Nursing students’ humanistic care ability directly determines their ability to work in the nursing profession in the future.^[3]^ Therefore, how to cultivate and improve the humanistic care ability of nursing students is the primary task in nursing education. At present, there are many researches on the humanistic care ability of nursing students, but fewer on the mediating effect of humanistic care ability.^[4,5]^

To our knowledge, the relationship between emotional intelligence, empathy and humanistic care ability has not been absolutely confirmed yet, especially among Chinese nursing students.^[6]^ Hence, the current study aimed to further investigate the relationship among emotional intelligence, empathy and humanistic care ability levels in nursing students and determine whether emotional intelligence mediates the relationship between empathy and humanistic care ability.

## 2. Background

It is the trend of The Times for medicine to return to humanity. In the outline of China’s Nursing career Development Plan issued by the National Health and Family Planning Commission, the word “humanities” was mentioned 3 times, and it was emphasized that humanistic education and quality education of nursing specialty should be strengthened in the reform and development of nursing education.^[7]^ Humanistic caring ability is a professional ability that nurses must possess and an important guarantee for providing high-quality nursing services. With the change of medical model and the development of holistic nursing, nursing practice increasingly emphasizes the respect for people and life.^[8]^ Caring is the basic element and core content of nursing, and providing people-oriented nursing services that care for the value of life is an essential professional moral quality for nursing staff today. Therefore, strengthening nursing humanistic care education not only accords with the trend of the integration of science education and humanistic education, but also meets the needs of the transformation of nursing education model.^[9]^ In China, nursing education focuses on professional knowledge and clinical skill training, which to some extent inhibits the development of humanistic care education. Therefore, to strengthen the humanistic care ability of nursing students is imperative.

Empathy is a kind of other-centered, selection of others’ opinions, experience others’ emotions, and then produce similar or consistent emotional experience and behavioral reaction with others, which has been proved has a positive correlation with humanistic care ability.^[10]^ Nurses with good empathy stand in the patient’s position to experience their psychological processes and can accurately evaluate their emotional needs, and then provide humanistic care.^[11]^ Empathy in clinical nursing practice plays an important role in improving patient satisfaction, alleviating conflict and reducing medical disputes. One study by Abe et al^[12]^ established emotional intelligence can improve nurses’ empathy. Emotional intelligence is an individual’s ability to perceive, understand and manage their own and other people’s emotions in different degrees, including the use of skills to regulate positive and negative emotions in the work environment, social environment and educational environment.^[13]^ Differences in these abilities will affect their adaptability to various environments. As a non-cognitive skill, emotional intelligence can help nurses manage situational stressors and make decisions in nursing work, reduce the negative impact of stress and emotion, and have a positive impact on the work quality and physical and mental health of nurses.^[14]^

With the increasing research on emotional intelligence of nursing students, studies have shown that emotional intelligence is significantly positively correlated with humanistic care ability, and improving emotional intelligence can improve humanistic care ability.^[15]^ Therefore, it is worthwhile to explore whether emotional intelligence could have a mediating effect on the relationship between empathy and humanistic care ability among student nurses.

The aim of the present study is to further explore the relationship between emotional intelligence, empathy and humanistic care ability among nursing students and further uncover the mediating role of emotional intelligence to reveal the internal mechanism of empathy on humanistic care ability, and provides evidence for improving the level of empathy and promoting their humanistic care ability.

## 3. Methods

### 3.1. Design and sample

This descriptive correlation study was designed to examine the relationship among emotional intelligence, empathy and humanistic care ability in nursing students. Stratified random sampling method was used to select nursing students of [removed for peer review]. The inclusion criteria were students major in nursing, willing to participant, and being able to complete the questionnaires. Finally 323 students participate in this study and included in our analysis.

### 3.2. Instruments

The general demographic questionnaire included age, gender, grade, residence, whether from single-child family, et al.

In this study, the Chinese version of caring ability Scale translated by Xu et al^[15]^ was used to evaluate students’ humanistic caring ability. The scale includes 37 items, which are divided into 3 dimensions of cognition, patience and courage. Each item is scored on a 7-point Likert scale ranging from 1 (not disagree) to 7 (totally agree). The total score ranges from 37 to 259, and the higher the score is, the stronger humanistic care ability is. The scale has good reliability and validity. The Cronbach’s α was 0.84 in the previous study^[16]^ and 0.88 in this study.

The 16-item emotional intelligence scale developed by Wong^[17]^ was used to measure nursing students’ emotional intelligence level. It consists of 4 subcategories, including assess self-emotion, assess other’ emotion, regulate emotion and apply emotion. Each item is scored on a 7-point Likert scale ranging from 1 (not disagree) to 7 (totally agree). Higher scores indicated higher emotional intelligence level. This scale has been widely used among Chinese students and has good reliability and validity. The Cronbach’s α was 0.94 in the previous study^[17]^ and 0.91 in this study.

The Jefferson Empathy Scale translated by Qiu et al^[18]^ was used to evaluate the empathy level of students. The scale consists of 20 items, including 3 dimensions of viewpoint selection, emotional nursing and perspective-taking. Each item is scored on a 7-point Likert scale ranging from 1 (not disagree) to 7 (totally agree). Higher scores indicated higher empathy level. This scale has been widely used among Chinese students and has good reliability and validity. The Cronbach’s α was 0.89 in the previous study^[18]^ and 0.86 in this study.

### 3.3. Data collection

The online survey link was send by the researcher to all nursing students who were willing to participate the study. All data were collected by the WenJuanXing program. In order to improve the accuracy of questionnaire response, the electronic questionnaire has been equipped with logic check, error reminder, automatic jump and other verification procedures.

### 3.4. Ethical considerations

This study was approved by the Ethics committee of Harbin Medical University. The researcher illustrated the aim of the study and promise that all data would be anonymized. Students can withdraw from the study at any time with any reason. An online informed consent form was completed by each participants.

### 3.5. Data analysis

The Statistical Packages for Social Sciences, version 25 (IBM Corp., Chicago, IL) was used to analyze the data. The mean and standard deviation of humanistic care ability of different ages and grades were analyzed by descriptive statistics, independent sample *t* test and one-way analysis of variance. Pearson’s correlation test was used to investigate the relationship among emotional intelligence, empathy, and humanistic care ability. The mediation effect test model of Bootstrap was used to test and analyze the mediating effect of emotional intelligence whether played a mediating role between empathy and humanistic care ability. A *P* value ≤ .05 was considered statistically significant.

## 4. Results

The average age of the participants was 19.52 ± 1.25 years with a range of 17 to 23 years. 282 participants (87.30%) were women. The residence in urban accounted for 60.68%. 184 participants (56.96%) were single-child family.

As shown in Table 1, the total score of humanistic care ability has significant differences among grades and major interests, however there was no significance among different gender, residence, only-child family and experience in being a student leader (*P* > .05).

**Table 1:** The difference between groups of humanistic care ability (N = 323).

Variables		Humanistic care ability score	t/F	*P*
Gender	Male	178.06 ± 20.12	0.46^a^	.61
	Female	180.83 ± 22.15		
Grade	Freshmen	176.36 ± 20.01	4.55^†^	.004
	Sophomore	177.63 ± 19.53		
	Junior	179.16 ± 23.76		
	Senior	188.19 ± 21.08		
Residence	Urban	180.35 ± 22.14	0.54^a^	.59
	Rural	178.80 ± 21.12		
Single-child family	Yes	181.25 ± 23.84	1.29^a^	.22
	No	177.97 ± 19.25		
Experience in being a student leader	Yes	181.43 ± 22.44	0.67^a^	.50
	No	179.44 ± 22.64		
Major matches the interests	Yes	185.10 ± 19.25	7.06^†^	<.01
	General	176.22 ± 20.73		
	No	176.05 ± 28.42		

a *t* test.

† One-way analysis of variance

The mean scores of emotional intelligence, empathy, and humanistic care ability were found to be 78.98 ± 14.97, 87.39 ± 11.58, and 179.60 ± 21.08, respectively. Furthermore, Table 2 showed the significant positive correlations between participants’ humanistic care ability and emotional intelligence (*R* = 0.62, *P* < .01) and empathy (*R* = 0.57, *P* < .01). Emotional intelligence had a significant positive correlation with empathy (*R* = 0.37, *P* < .01).

**Table 2:** Means and correlation coefficients of the variables (N = 323).

Variables	Mean ± SD	Emotional intelligence (r)	Empathy (r)
Emotional intelligence	78.98 ± 14.97		
Empathy	87.39 ± 11.58	0.37*	
Humanistic care ability	179.62 ± 21.08	0.62*	0.57*

SD = standard deviation.

* *P* < .01.

**Figure 1.**
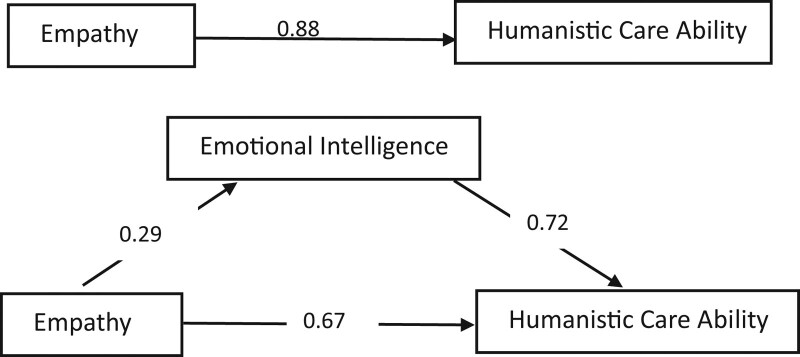
Model diagram of the mediating effect of emotional intelligence on empathy on humanistic care ability.

In the regression analysis, we set humanistic care ability as the dependent variable and empathy as the independent variable and emotional intelligence as mediating variable. The results were shown in Table 3 and Figure 1. The results showed that emotional intelligence mediated the effects of empathy on humanistic care ability, with an explanatory power of 23.86%.

**Table 3:** Mediating analyze of emotional intelligence on empathy on humanistic care ability (N = 323).

	β	SE	95%CI		Account
			Lower	Upper	
Total effect	0.88	0.06	0.75	0.99	
Direct effect	0.67	0.05	0.55	0.78	76.14%
Indirect effect	0.21	0.05	0.11	0.32	23.86%

CI = confidence interval, SE = standard error.

## 5. Discussion

The current study, which aimed to investigate the relationship between emotional intelligence, empathy, and humanistic care ability in nursing students, found that emotional intelligence mediated the relationship between empathy and humanistic care ability.

In this study, the humanistic care ability of undergraduate midwifery students was poor. This finding is similar to relevant results of Chinese nursing students,^[6,7]^ but far lower than the level of humanistic care ability in foreign counties.^[19]^ An international cross-cultural study showed that the humanistic care ability of nursing students in China was lower than that in Slovenia, Russia, the United States and other countries.^[20]^ This may be related to the differences in medical education models in different countries. China’s nursing education is still in its infancy, and more attention is paid to cultivating students’ knowledge and skills. Although humanistic caring ability is defined as the ability that nursing students should be equipped, it is often an additional state in the implementation of education and fails to really integrate into the whole education. In addition, the nursing major is more practical and requires students to deeply understand the professional characteristics in clinical work, so as to experience the humanistic care beyond the routine courses.^[21]^ However, for the nursing education in China, the proportion of clinical practice is relatively small, which may be one of the factors leading to the low humanistic care ability of nursing students in China. The results of this study showed that grade has a statistically significant impact on humanistic care ability, the higher the grade, the higher humanistic care ability was. With the increase of age, nursing students improve their cognitive level and have a positive effect on their own ability in all aspects. At the same time, whether the major is consistent with the interest also affects their humanistic care ability. Nursing students who are interested in nursing can actively acquire professional nursing knowledge and actively participate in nursing practice, and know how to self-reflect on the process of practice, and have a deeper understanding and experience of nursing.^[22]^ It is the mission of nursing education to cultivate the humanistic care ability of nursing students, therefore, it is necessary to explore the multidimensional factors affecting the humanistic care ability of nursing students, so as to take effective measures to improve their humanistic care ability.

The American Association of Colleges of Nursing clearly points out that empathy is a key component of nursing and important to nursing education.^[23]^ Empathy is an ability or skilled skill that can be learned and developed through education and practice.^[24]^ The current study demonstrated significant correlations among empathy and humanistic care ability in nursing students, which consistent with those of previous studies.^[6,25]^ If nursing students can better do perspective-taking, understand the importance of patients and nursing measures, and then nursing students will have a better understanding of professional responsibilities and obligations, but also have the courage and patience to relieve the pain caused by the disease, which improves the humanistic care ability of nursing students. This suggests that nursing educators should pay attention to cultivate the ability of empathy to improve humanistic care ability of nursing students.^[6]^ Empathy is the precondition of implementing humanistic care nursing, should also be as the important part of humanistic care education and training.^[26]^ In the previous study, researcher suggested that the cultivation of students’ empathy ability should be emphasized in relevant core courses, using simulation reflection, role playing, the real case analysis, etc.^[6]^ At the same time, students should be encouraged to participate in more social activities, encourage students to learn from life experience, examine the needs and feelings of others, and thus improve empathy.^[27]^

This study showed that the emotional intelligence was positively correlated with the humanistic care ability, indicating that nursing students with high emotional intelligence level have the high humanistic care ability. When nursing students can accurately perceive the emotional changes of the other side, and express their emotions through language and expression, and can reasonably control themselves in different situations, they can pay attention to understand others, the needs of others, and build a harmonious nurse-patient relationship.^[28]^ Emotional intelligence can stimulate students’ inner emotions into practical actions, nurse educators should strengthen the education and cultivation of emotional intelligence for nursing students, so as to transform their emotions into humanistic care ability. For example, some researchers implemented experiential teaching based on the cultivation of emotional intelligence among nursing students, and cultivated emotional intelligence through emotional experience activities (scenario simulation, role playing, etc), which achieved significant intervention results.^[29]^

Moreover, our results also confirmed that emotional intelligence affected the students’ humanistic care ability, which also mediated the relationship between empathy and humanistic care ability. Nursing students with high emotional intelligence have relatively high ability to assess, adjust and use emotions of themselves and others, they have high interpersonal communication skills and the ability to use emotions to achieve goals and are more willing to take the initiative care for patients.^[30]^ Previous studies showed that emotional intelligence was not innate, but can be improved through training.^[31]^ It suggests that the emotional intelligence of nursing students can be enhanced so as to stimulate the individual’s inner emotion, predict and understand the emotions and behaviors of others better, and promote the formation of nursing students’ humanistic care ability.^[6]^ Therefore, to enhance the humanistic care ability of nursing students, nursing educators should not only improve their empathy, but also pay attention to cultivate emotional intelligence of nursing students.

### 5.1. Limitations

Although this study identified the relationship of nursing students’ emotional intelligence, empathy and humanistic care ability, there were still limitations to this study. Firstly, the participants of this study were recruited from one university, readers need to raise caution when applying our findings to nursing students from other countries or from different cultural backgrounds. Further studies should be conducted on larger samples in different cultures. Moreover, the self-report nature of the measures mean that responses are subject to bias and socially desirable responding.

## 6. Conclusions

The results in this study suggested that the humanistic care ability of nursing students was poor and was positively associated with emotional intelligence and empathy. Moreover, this study confirmed the mediating effect of emotional intelligence on empathy and humanistic care ability in nursing students. The results of this study could help nurse educators better understand the relationship between empathy, emotional intelligence and humanistic care ability. It is suggested that nursing educators should ought to assist students in cultivating emotional intelligence as a means to improve the empathy and thus enhance the humanistic care ability.

## Acknowledgment

All authors were grateful for all the participants and all student counselors in this study for their corporation.

## Author contributions

**Conceptualization:** Meng Lina, Guan Qin, Li Yang.

**Data curation:** Meng Lina, Guan Qin, Li Yang.

**Formal analysis:** Li Yang.

**Funding acquisition:** Meng Lina, Guan Qin.

**Investigation:** Meng Lina, Guan Qin, Li Yang.

**Methodology:** Meng Lina, Guan Qin, Li Yang.

**Project administration:** Li Yang.

**Validation:** Li Yang.

**Visualization:** Li Yang.

**Writing – original draft:** Meng Lina, Guan Qin, Li Yang.

**Writing – review & editing:** Meng Lina, Guan Qin, Li Yang.
